# Elevated levels of exogenous prolactin promote inflammation at the maternal-fetal interface via the JAK2/STAT5B signaling axis

**DOI:** 10.3389/fimmu.2024.1496610

**Published:** 2024-12-23

**Authors:** Alycia Williams, Daniel J. Hossack, Nia Thompson, Yul Eum Sim, Cristina Wilson, Viviane Schuch, Tiffany Hailstorks, Rana Chakraborty, Erica L. Johnson

**Affiliations:** ^1^ Department of Microbiology, Biochemistry, and Immunology, Morehouse School of Medicine, Atlanta, GA, United States; ^2^ Department of Natural Science, Greensboro College, Greensboro, NC, United States; ^3^ Clinical Research Center, Morehouse School of Medicine, Atlanta, GA, United States; ^4^ Department of Gynecology and Obstetrics, Emory University, Atlanta, GA, United States; ^5^ Division of Pediatric Infectious Diseases, Department of Pediatrics, Miller School of Medicine, University of Miami, Miami, FL, United States; ^6^ Department of Obstetrics and Gynecology, Morehouse School of Medicine, Atlanta, GA, United States

**Keywords:** prolactin, STAT5, placenta, decidua, pregnancy, inflammation, LPS, SOCS

## Abstract

The placenta is a unique organ with various immunological and endocrinological roles that modulate maternal and fetal physiology to promote maternal-fetal tolerance, pregnancy maintenance, and parturition at term. During pregnancy, the hormone prolactin (PRL) is constitutively secreted by the placenta and is necessary for implantation, progesterone support, fetal development, and overall immune modulation. While PRL is essential for pregnancy, studies suggest that elevated levels of serum PRL (hyperprolactinemia) are associated with adverse pregnancy outcomes, including miscarriage, preterm birth, and preeclampsia. However, there is a lack of mechanistic studies to support these observations. Here we investigated the impact of elevated levels of PRL on placental cells and evaluated PRL effects on the JAK2/STAT5 inflammatory signaling cascade. Elevated levels of exogenous PRL enhances PRL and PRL-receptor expression, along with JAK2/STAT5 signaling in primary decidual mononuclear cells and the placental trophoblast cell line, JEG-3. Following PRL exposure, the STAT5 isoform, STAT5B, is preferentially activated and there is a significant upregulation in the secretion of pro-inflammatory cytokines, IL-6 and IL-1β. This inflammatory cascade is supported via PRL-induced reduction of SOCS1 and SOCS2. Furthermore, LPS exacerbates PRL expression and JAK2/STAT5 signaling, leading to increased secretion of IL-6 and TNF-α. These results highlight the inflammatory roles of elevated PRL at the maternal-fetal interface, underscoring the need for further mechanistic studies to elucidate its functions in pregnancy.

## Introduction

Pregnant women are immunologically unique because their immune system is influenced by signals originating from the placenta (1). These signals promote homeostasis of hormones and cytokines throughout gestation, which actively influence maternal and fetal physiology including tolerance of the HLA-discordant fetus, maintenance of pregnancy, and parturition at term (1). The placenta is a dynamic endocrinologic organ, which secretes gestation-dependent levels of prolactin and growth hormone family, steroid hormones, and neuroactive hormones. In a normal, healthy pregnancy the synthesis of T helper 2 (Th2) cytokines is predominant, while immune cells that reside at the maternal-fetal interface actively suppress Th1 responses (2). Disturbances at the maternal-fetal interface that disrupt this balance can result in pregnancy loss or pregnancy complications including preeclampsia and fetal growth restriction (3, 4). Numerous studies have associated elevated levels of prolactin in maternal serum (hyperprolactinemia), amniotic fluid, or cervicovaginal secretions with pregnancy loss and/or preterm birth (5–11). However, there is a lack of mechanistic studies to support this observation.

Prolactin (PRL) is a pleiotropic hormone secreted in the pituitary gland, through the hypothalamic–pituitary–adrenal axis. It is also produced in extra-pituitary locations, such as the decidua, ovary, prostate, mammary gland, adipose tissue, brain, and immune cells (12). Extra-pituitary PRL has mainly autocrine and paracrine rather than endocrine effects due to different bioactivity and molecular weight from pituitary PRL (13). PRL has critical functions for reproduction such as the support of progesterone production, successful implantation, fetal development, and immunologic modulation (14). Prolactin is best known for its role in inducing and maintaining lactation, however during pregnancy, PRL plays an important role for placental growth and angiogenesis (15, 16).

PRL acts through specific receptors belonging to the cytokine receptor super-family, which are also receptors for leptin, erythropoietin, colony-stimulating factor, interleukin-6 (IL-6) among others (17–19). PRL receptors are broadly expressed in different endocrine and target tissues as well as immune cells including lymphocytes, monocytes, macrophages, granulocytes, microglia, natural killer (NK) cells, and thymic epithelial cells for controlling immune responses (20). The principal signaling pathway activated by PRL receptor interaction is the JAK/STAT pathway. PRL binding induces the dimerization of the receptor and activation of Janus Kinase 2 (JAK2), a kinase constitutively associated with the PRL receptor (21). The downstream signaling molecules activated by this receptor family have not been completely elucidated. However, it has been shown that JAK2 phosphorylates multiple tyrosine residues of the PRL receptor and enables the binding of signal transducer and activator of transcription (STAT) 5 proteins (22). Tyrosine-phosphorylated STAT5 dissociates from the receptor, dimerizes, and translocates into the nucleus where it binds to the promoters of target genes. Along with JAK2/STAT5, PRL receptor binding can activate downstream signaling cascades involving mitogen-activated protein kinase (MAPK), extra-cellular signal regulated kinase 1/2 (ERK1/2), and phosphoinositide 3-kinase (PI3K) intracellular pathways. The signaling of PRL through the JAK2-STAT5 pathway can be inhibited by several proteins. For example, cytokine signal suppressor proteins 1 and 3 (SOCS1 and SOCS3) regulate PRL signaling and its receptor. These proteins contain an Src-homologous domain (SH2), and a C-terminal SOCS box. The SH2 domain allows these proteins to bind to phosphorylated tyrosine residues, thereby inhibiting the JAK2 signaling pathway (23, 24). However, the role of this process in the regulation of the STAT5 proteins is not clear (23, 25).

PRL has been hypothesized to function as a cytokine with tissue-specific immunomodulatory activities. Studies suggest that prolactin plays a key role in regulating the equilibrium between pro- and anti-inflammatory modulators at the maternal-fetal interface. For example, PRL treatment was shown to induce IL-1β, interferon gamma (IFN-γ) and tumor necrosis factor alpha (TNF-α) release by murine peritoneal macrophages *in vitro* (26). In addition, studies show that PRL can downregulate IL-1β and TNF-α in cultured chorioamniotic membranes (27, 28). In contrast, other work suggests that PRL can alter Th2 environments, promoting IL-6 and IFN-γ secretion (29, 30). In macrophages and dendritic cells, PRL has been associated with abnormal innate and adaptive immune responses, along with the increased production of IL-6, IL-12, TNF-α, and IL-1β (31, 32). Taken together, PRL may have dual inflammatory and anti-inflammatory effects depending on the underlying pathophysiological conditions. However, the role of PRL at the maternal-fetal interface is unclear and there is a lack of mechanistic studies to describe its role. Here we evaluate the impact of PRL exposure and signaling on primary decidual macrophages and the placental trophoblast, which are key immune mediators at the maternal-fetal interface. Specifically, we analyze the effects of different concentrations of PRL on JAK2/STAT5 signaling and the secretion of TNF-α, IL-1β, IL-6, and IL-10. We also use LPS to mimic an underlying inflammatory condition and explored its ability to impact PRL gene expression and modulation of the PRL receptor (PRLR) signaling pathway. Our data show that exogenous treatment of PRL induces elevated expression of PRL and PRL receptor in primary decidual macrophages and the placental trophoblast cell line, JEG-3. This upregulation is associated with robust JAK2/STAT5 signaling and increased secretion of pro-inflammatory cytokines. Interestingly, PRL signaling preferentially induced the activation of the STAT5B isoform, compared to STAT5A. Our findings also suggest that LPS exacerbates PRL expression and downstream activity in placental cells. *In sum*, we show that elevated levels of PRL at the maternal-fetal interface may promote an inflammatory milieu, associated with JAK2/STAT5B signaling and underlying inflammatory conditions may exacerbate PRL secretion in placental trophoblasts, associated with inflammation and adverse pregnancy outcomes.

## Materials and methods

### Ethics statement

Human term placentae (>37 weeks gestation) were collected from hepatitis B, HIV-1 seronegative women (>18 years of age) immediately after elective caesarean section without labor from Emory Midtown Hospital, Atlanta, GA and Grady Memorial Hospital, Atlanta, Georgia. This study was approved by the Morehouse School of Medicine Institutional Review Board (1964407-8) and the Emory University Institutional Review Board (IRB 000217715). Written informed consent was acquired from all donors before sample collection. Samples were de-identified before primary decidual macrophage cell isolation.

### Placental dissection and decidual macrophage isolation

Decidual macrophages were isolated from the maternal side of the placenta as previously described (33). Briefly, the decidual basalis tissue was thoroughly washed and mechanically dispersed in Hank’s balanced salt solution (HBSS) to minimize peripheral blood contamination. The minced tissue was re-suspended in complete medium containing 1 mg/ml collagenase A (Worthington Biochemical, Lakewood, NJ, USA) and 0.2 mg/ml of DNAse I (Sigma-Aldrich, St. Louis, MO, USA) and incubated in a shaking water bath at 37°C for 1 hour. The digested tissue was washed with PBS and passed through gauze and a 70 µm cell strainer (BD-Falcon Biosciences, Lexington, TN, USA). The mononuclear cell population was isolated by density gradient centrifugation on Histopaque-1077 (Sigma-Aldrich). CD14^+^ Magnetic Cell Sorting was performed using anti-CD14 magnetic beads (Miltenyi Biotech, Bergisch Gladbach, Germany) as recommended by the manufacturer. On average, the purity was >95%. After isolation, decidual macrophages were cultured in complete RPMI medium consisting of 1x RPMI (Corning Cellgro, Corning, NY, USA), 10% FBS (Optima, Atlanta Biologics, Atlanta, GA, USA), 2mM L-glutamine (Corning), 1mM sodium pyruvate (Corning), 1x Non-essential Amino Acids (Corning), 1x antibiotics (penicillin, streptomycin, amphotericin B; Corning) at 37°C and 5% CO2.

### Cell culture

The choriocarcinoma-derived JEG-3 cell-line (ATCC HTB-36, Manassas, VA, USA), was grown as recommended by ATCC: Minimum Essential Medium Eagle’s medium supplemented with 10% FBS, 2 mM of glutamine, 50 IU/ml of penicillin and 50 IU/ml of streptomycin. Cells were detached using trypsin, counted, and then seeded at 400,000 cells/mL in 24-well plates (500 μL by well). The decidual macrophages and JEG-3 cells were treated for 15 min, 24 hours and 48 hours with the following as indicated, following resuspension per the manufacturer’s instructions: 10ng/mL and 100ng/mL of Prolactin (R&D Systems, Minneapolis, MN, USA) and 5μg/mL of LPS (Invitrogen, Carlsbad, CA, USA).

### RNA isolation and quantitative RT-PCR

Messenger RNA (mRNA) was extracted using the RNeasy kit (Qiagen, Hilden, Germany). The complementary DNA was transcribed using QuantiTect RT kit (Qiagen). JEG-3 and decidual macrophage gene expression was quantified by qRT-PCR using QuantiTect SYBR Green PCR Kits (Qiagen) with specific primers for host and integrated viral genes (**Supplementary Table S1**). Delta cycle threshold (ΔCt) values from the calibrator and experimental groups were measured by subtracting Ct values from target vs the housekeeping transcript, β-actin. Gene expression data are represented as ΔCt values or as fold change relative to paired, time-matched, untreated controls (gene expression normalized to β-actin − ΔΔCt method).

### ELISA

IL-6, IL-1β, and TNF-α concentrations in the supernatants of treated decidual macrophages, JEG-3 cells, and accompanying controls were assessed in duplicate using ELISA MAX Deluxe Set Human Kits (Biolegend, San Diego, CA, USA) according to the manufacturers’ instructions. Plates were read on a BioTek Synergy H1 plate reader and concentrations were determined by comparison to a standard curve.

### Immunoblotting

Cells were lysed in radio-immunoprecipitation assay buffer (ThermoFisher, Waltham, MA, USA) with protease inhibitors (Roche, Basel, Switzerland). Samples were subjected to denaturing sodium dodecyl sulfate–polyacrylamide gel electrophoresis. Gels were blotted on nitrocellulose membranes (GE Healthcare, Chicago, IL, USA). After blocking with buffer (Li-Cor; Lincoln, NE, USA), the membrane was incubated with the following primary antibodies overnight at 4°C (**Supplementary Table S2**): rabbit anti-STAT5A (131791AP), rabbit anti-pSTAT5A (801151RR), rabbit anti-STAT5B (510722AP), rabbit anti-pSTAT5B (ab52211), and rabbit anti-GAPDH (D16H11) [Proteintech, Rosemont, IL, USA; Cell Signaling Technology, Danvers, MA, USA; Abcam, Cambridge, UK]. Next, the membrane was incubated with goat anti-rabbit, HRP-linked secondary antibodies (7074S), washed, and then incubated in ImmunoCruz Western blotting luminol reagent (Santa Cruz Biotechnogy, Dallas, TX, USA). Images were captured with the Invitrogen iBright CL1500 Imaging System. Densitometry was performed using ImageJ software (34). Bands of interest were normalized to GAPDH.

### Statistical analysis

All figures are representative of at least 3 independent experiments and 8 individual donors. For statistical testing and graphical depiction, Prism10 (GraphPad Software, Boston, MA, USA) was used. Data were analyzed using a parametric one-way ANOVA using Dunnett’s multiple comparisons test or a two-way ANOVA using Sidak’s multiple comparisons test. Analyses were corrected for multiple comparisons by controlling the false discovery rate. Discovery was determined using the two-stage linear step-up procedure of Benjamini, Krieger and Yekutieli, with Q = 5%, without assuming consistent SD (35). Differences were defined as significant when p ≤.05. Further experimental statistical details are described in the figure legends. ****p ≤ 0.0001; ***p ≤ 0.001; **p ≤ 0.01; *p ≤ 0.05; ns, nonsignificant.

## Results

### Exogenous PRL induces PRL and PRL-receptor mRNA expression in placental trophoblasts and decidual macrophages

**Figure 1:**
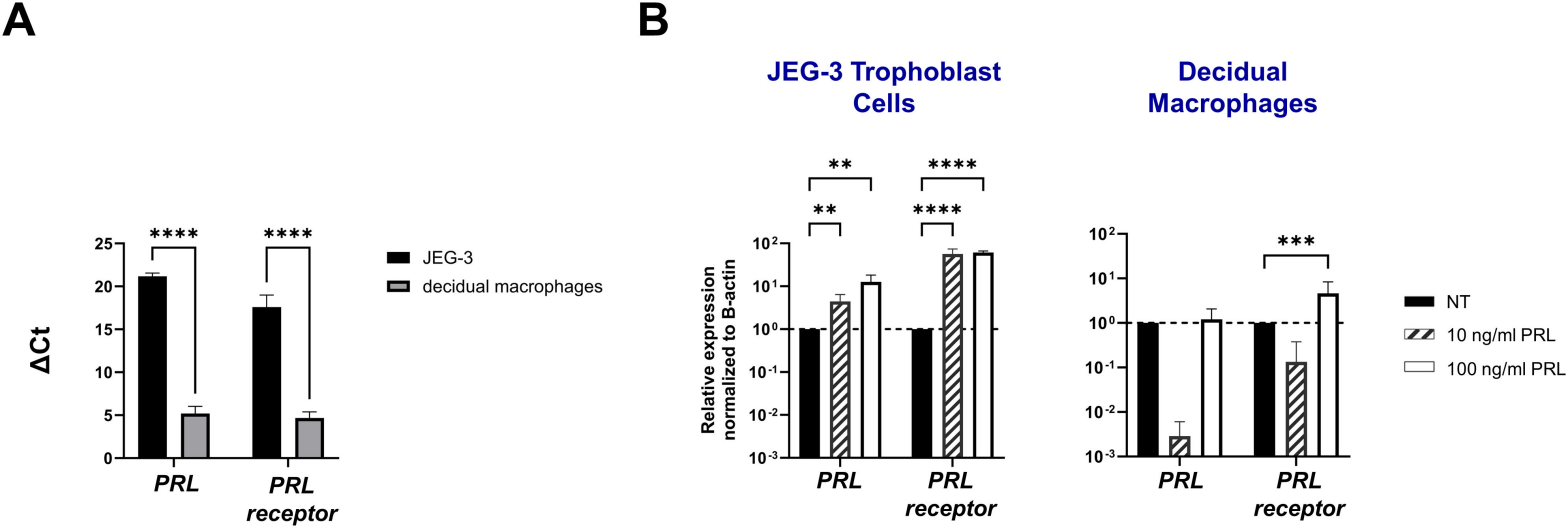
Exogenous PRL induces PRL and PRL-receptor mRNA expression in placental trophoblasts and decidual macrophages. **(A)** JEG-3 trophoblast cells were cultured and human decidual macrophages were isolated from freshly obtained placentae. Messenger RNA levels were measured by qRT-PCR to determine the basal transcription of PRL and the PRL receptor. Gene expression data are represented as ΔCt (normalized to β-actin). Data shown represent individual donors (n=8) analyzed by two-way ANOVA using Sidak’s multiple comparisons test. ****p < 0.0001 indicates significance between JEG-3 and decidual macrophages. **(B)** JEG-3 trophoblast cells and decidual macrophages were treated with 10 ng/ml of PRL, 100 ng/ml of PRL, or left untreated for 24 hours. Messenger RNA levels were measured by qRT-PCR to determine the relative expression of PRL and the PRL receptor. Gene expression data are represented as fold change normalized to β-actin (ΔΔ cycle threshold method). ****p< 0.0001, ***p <.001, and **p <.01 indicate significance between the untreated (NT) cells and the PRL treated cells.

To study the paracrine activity of PRL in placental cells, we measured the mRNA expression of PRL and PRL-receptor in JEG-3 trophoblast cells and decidual macrophages treated with increasing concentrations of human PRL. At a basal level, decidual macrophages exhibited significantly lower cycle threshold (Ct) values (± standard error [SE]) for *prolactin* (5.2 ± 2.32) and *prolactin-receptor* (4.68 ± 2.03) transcripts compared to JEG-3 cells, which had higher Ct values for *prolactin* (21.18 ± 1.07) and *prolactin-receptor* (17.584 ± 4) (**Figure 1A**). These lower Ct values indicate higher basal expression levels of PRL and PRL-receptor genes in decidual macrophages compared to JEG-3 cells. Further analysis by quantitative reverse transcription-PCR (qRT-PCR) showed a significant increase in PRL expression in JEG-3 cells treated with 10 ng/mL (p<0.0033) and 100 ng/mL PRL (p<0.0010) (**Figure 1B**). In contrast, in decidual macrophages, the lower concentration (10 ng/mL) of PRL induced a downregulation in PRL mRNA expression, while 100 ng/mL of exogenous PRL had no impact on PRL expression. In addition, PRL treatment significantly increased PRL-receptor expression in both JEG-3 cells and decidual macrophages (p<0.0001). These data suggest that elevated levels of exogenous PRL induce the production of PRL in trophoblast cells, which is associated with elevated PRL-receptor expression and paracrine signaling at the maternal-fetal interface.

### Prolactin exposure induces JAK2/STAT5 signaling in placental cells

**Figure 2:**
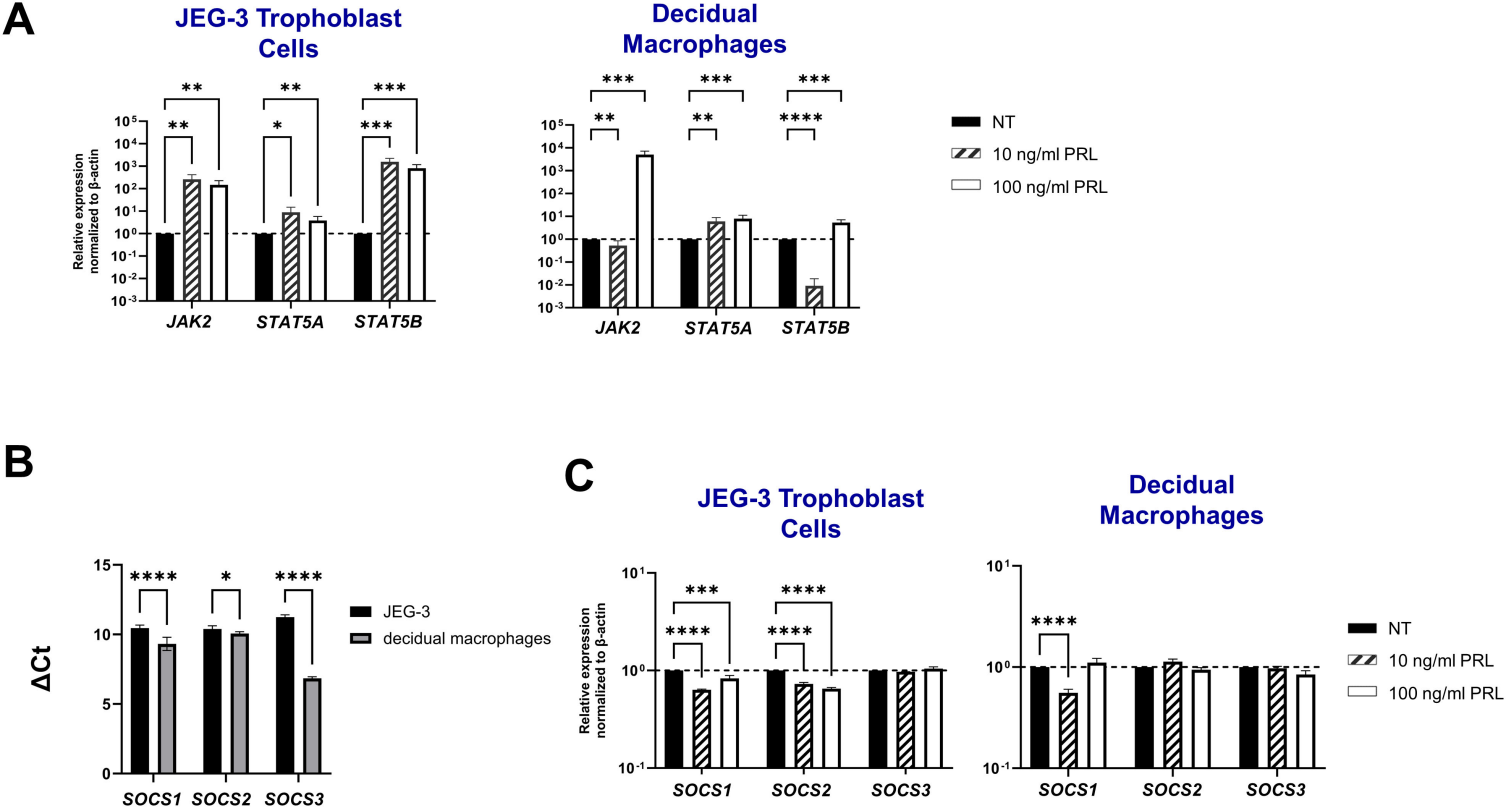
Prolactin treatment induces JAK2/STAT5 signaling in placental cells. **(A)** JEG-3 trophoblast cells and primary decidual macrophages were treated with 10 ng/ml of PRL, 100 ng/ml of PRL, or left untreated for 24 hours. The JAK2/STAT5 signaling pathway was evaluated by measuring messenger RNA levels of JAK2, STAT5A, and STAT5B by qRT-PCR. Gene expression data are represented as fold change relative to time-matched, untreated cells (gene expression normalized to β-actin – ΔΔ cycle threshold method). Data shown are expressed as the mean ± standard error of biological triplicates from 8 individual donors analyzed by two-way ANOVA using Sidak’s multiple comparisons test. ****p< 0.0001, ***p <.001, **p <.01, and *p <.05 indicate significance between the untreated (NT) cells and the PRL treated cells. **(B)** Messenger RNA levels were measured by qRT-PCR to determine the basal transcription of the SOCS family of proteins (SOCS1, SOCS2, and SOCS3), which are negative-feedback inhibitors of the JAK/STAT pathway. Gene expression data are represented as ΔCt (normalized to β-actin). Data shown represent individual donors (n=8) analyzed by two-way ANOVA using Sidak’s multiple comparisons test. ****p < 0.0001 and *p <.05 indicate significance between JEG-3 and decidual macrophages. **(C)** JEG-3 trophoblast cells and primary decidual macrophages were treated with 10 ng/ml of PRL, 100 ng/ml of PRL, or left untreated for 24 hours. SOCS1, SOCS2, and SOCS3 were evaluated by qRT-PCR. Gene expression data are represented as fold change relative to time-matched, untreated cells (gene expression normalized to β-actin – ΔΔ cycle threshold method). Data shown are expressed as the mean ± standard error of biological triplicates from 8 individual donors analyzed by two-way ANOVA using Sidak’s multiple comparisons test. ****p< 0.0001 indicates significance between the untreated (NT) cells and the PRL treated cells.

To characterize PRL-signaling in placental trophoblasts and decidual macrophages, we measured mRNA concentrations of key signaling molecules: JAK2, STAT5A, STAT5B, SOCS1, SOCS2, and SOCS3. Our results demonstrate that exogenous treatment with PRL robustly activates the JAK/STAT pathway in these cells. Specifically, both low (10 ng/mL) and high (100 ng/ml) concentrations of PRL significantly increased the expression of JAK2, STAT5A, and STAT5B in JEG-3 cells. In decidual macrophages, only the higher concentration of PRL (100 ng/ml) induced the upregulation of JAK2, STAT5A, and STAT5B, while the lower concentration (10 ng/ml) caused a significant downregulation of JAK2 and STAT5B mRNA expression (**Figure 2A**).

We investigated expression of the suppressors of cytokine signaling (SOCS) family, known inhibitors of the JAK2 signaling pathway (23, 24), to explore their potential role at the maternal-fetal interface. Our analysis revealed that at a constitutive level, decidual macrophages exhibited significantly lower Ct values (± SE) for *SOCS1* (9.321 ± 0.17), *SOCS2* (10.072 ± 0.04) and *SOCS3* (6.847 ± 0.038) transcripts compared to JEG-3 cells (**Figure 2B**). These lower Ct values indicate higher basal expression levels of SOCS proteins in decidual macrophages. Additionally, we found that exogenous PRL treatment (10 ng/ml and 100 ng/ml) significantly reduced mRNA expression of SOCS1 (p<0.0001 and p=0.0009, respectively) and SOCS2 (p<0.0001) in JEG-3 cells; with no effect on SOCS3 expression. Similarly, PRL significantly downregulated SOCS1 mRNA expression in decidual macrophages (p<0.0001). These findings suggest that PRL robustly activates the JAK2/STAT5 pathway at the maternal-fetal interface, potentially through targeted reduction of SOCS1 and/or SOCS2 expression.

### Exogenous treatment with PRL induces the secretion of pro-inflammatory cytokines by placental cells

**Figure 3:**
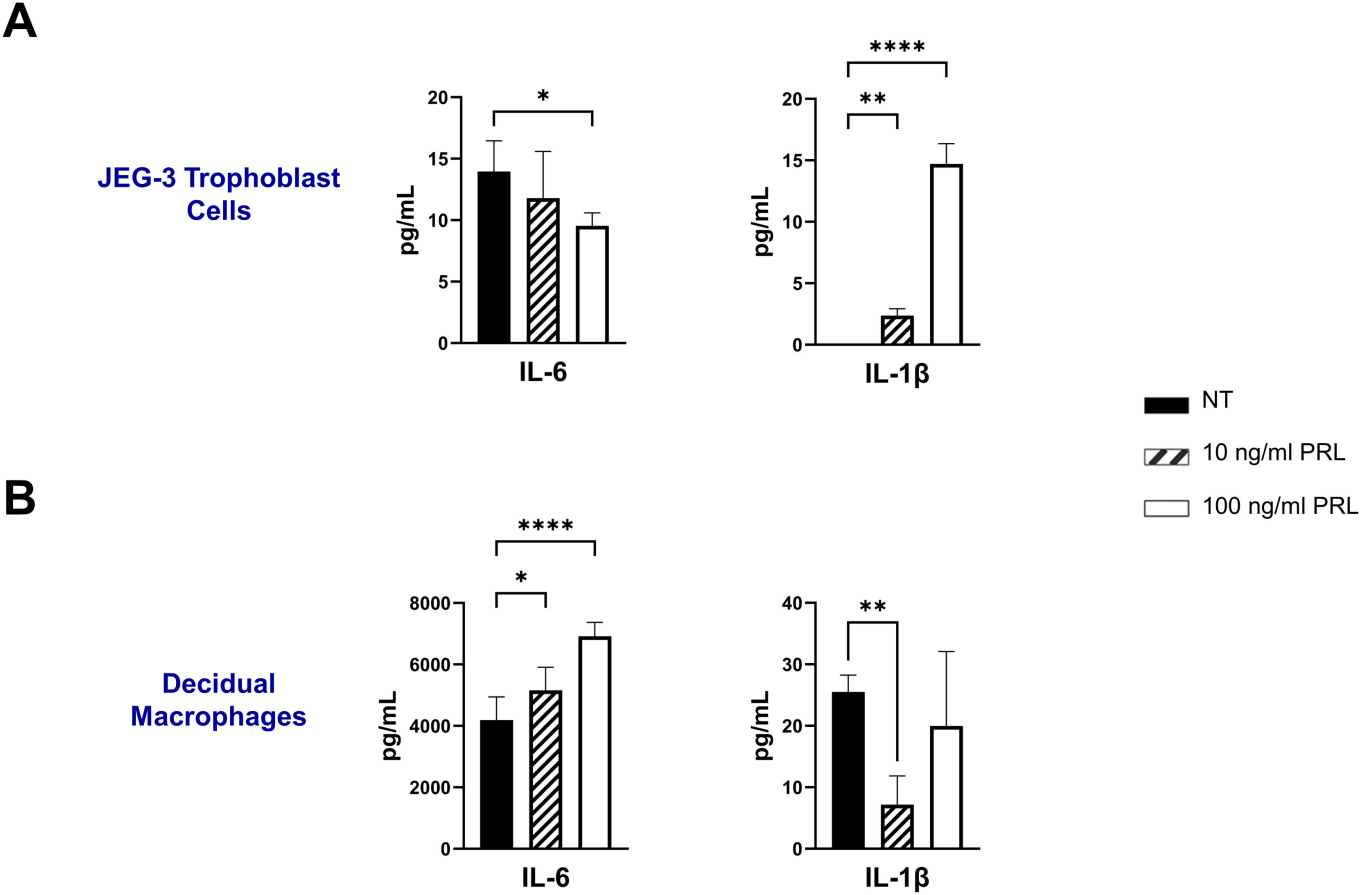
Exogenous treatment with PRL induces the secretion of pro-inflammatory cytokines by placental cells. JEG-3 trophoblast cells and primary decidual macrophages were treated with 10 ng/ml of PRL, 100 ng/ml of PRL, or left untreated. Quantification of inflammatory cytokines, IL-6 and IL-1β, were determined in the supernatants by ELISA following 48 hours of *in vitro* culture. All values are represented as pg/mL. Data shown are expressed as the mean ± standard error of biological triplicates from 8 individual donors analyzed by one-way ANOVA using Dunnett’s multiple comparisons test. ****p< 0.0001, **p <.01, and *p <.05 indicate significance between the untreated (NT) cells and the PRL treated cells.

Numerous hormones and cytokines signal via the JAK/STAT pathway, which plays a crucial role in mediating inflammatory responses and regulating diverse cellular processes, including cellular proliferation, survival, development, and apoptosis (36). We measured the release of inflammatory cytokines (IL-6, TNF-α, and IL-1β) following exogenous treatment with PRL (10 ng/mL or 100 ng/mL) in JEG-3 cells and decidual macrophages. Our findings show that decidual macrophages constitutively secrete higher levels of IL-6 compared to JEG-3 cells (4193.29 ± 751.39 vs 13.96 ± 2.49 [pg/ml]) including at basal levels (25.53 ± 2.71 pg/ml) without any treatment, while JEG-3 cells do not secrete IL-1β. Following PRL treatment, IL-6 secretion was significantly increased in decidual macrophages (p=0.0443), with a more pronounced effect at the higher PRL concentration (100 ng/ml, p<0.0001). In contrast, JEG-3 cells treated with 100 ng/ml PRL significantly downregulated IL-6 secretion (p=0.0228). PRL treatment inhibited IL-1β secretion in decidual macrophages, while it significantly upregulated IL-1β secretion in JEG-3 cells (**Figures 3A, B**). Interestingly, neither JEG-3 nor decidual macrophages secreted TNF-α at basal levels or in the response to PRL treatment. These data suggest that PRL exposure differentially regulates the expression of proinflammatory cytokines by immune cells at the maternal-fetal interface.

### LPS upregulates PRL-receptor expression and signaling at the maternal-fetal interface

**Figure 4:**
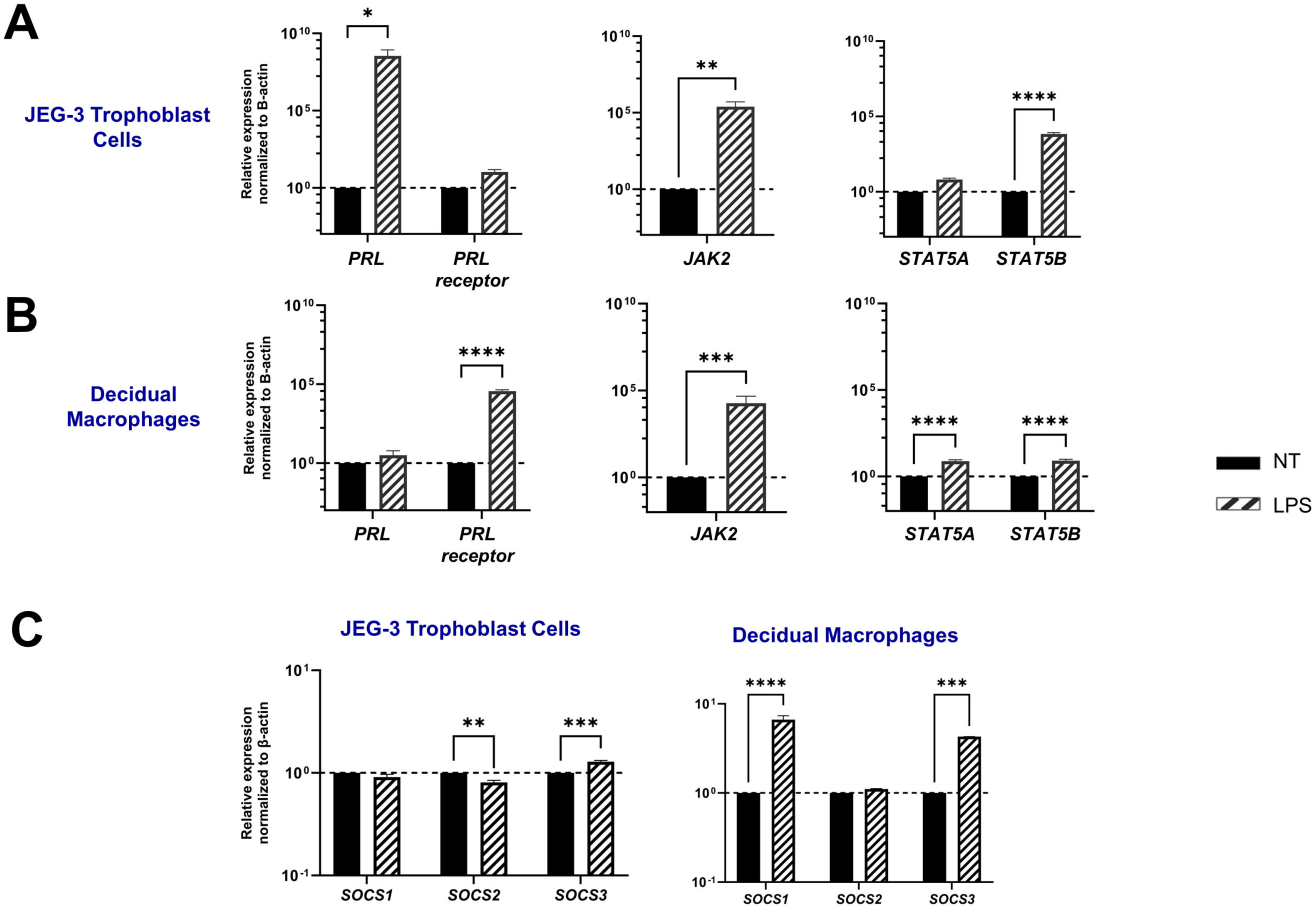
LPS upregulates PRL-receptor expression and signaling at the maternal-fetal interface. **(A)** JEG-3 cells and decidual macrophages were treated with 5 μg/ml of LPS or left untreated for 24 hours. Messenger RNA levels were measured by qRT-PCR to determine the relative expression of PRL, PRL receptor, JAK2, STAT5A, and STAT5B. Gene expression data are represented as fold change normalized to β-actin (ΔΔ cycle threshold method). ****p< 0.0001, ***p <.001, **p <.01, and *p <.05 indicate significance between the untreated (NT) cells and the LPS treated cells. **(B)** Under similar conditions, the SOCS family of proteins (SOCS1, SOCS2, and SOCS3), which are negative-feedback inhibitors of the JAK/STAT pathway, were evaluated by qRT-PCR. Gene expression data are represented as fold change relative to time-matched, untreated cells (gene expression normalized to β-actin – ΔΔ cycle threshold method). Data shown are expressed as the mean ± standard error of biological triplicates from 8 individual donors analyzed by two-way ANOVA using Sidak’s multiple comparisons test. ****p< 0.0001, ***p <.001, and **p <.01 indicate significance between the untreated (NT) cells and the LPS treated cells.

Bacterial lipopolysaccharide (LPS) has been shown to enhance serum prolactin levels in animal models (37). In addition, elevated PRL levels and gram-negative bacteria that produce LPS have both been independently linked to miscarriage and adverse pregnancy outcomes (7, 38). Here we assess PRL production and signaling following LPS exposure in JEG-3 cells and decidual macrophages. Our findings reveal that LPS significantly upregulates PRL mRNA expression in placental trophoblasts (p=0.0284) and PRL-receptor expression in decidual macrophages (p<0.0001) (**Figures 4A, B**). Furthermore, LPS was found to impact PRL signaling, by upregulating JAK2 mRNA expression in both JEG-3 cells (p=0.0022) and decidual macrophages (p=0.0002). In decidual macrophages, LPS treatment also significantly increased the mRNA expression of STAT5A and STAT5B, whereas in JEG-3 cells, only STAT5B was elevated.

SOCS proteins are key mediators in the negative regulation of the JAK-STAT pathway. Interestingly, our study shows that LPS has a differential impact on placental trophoblast cells compared to decidual macrophages. In the JEG-3 cells, LPS treatment did not significantly affect SOCS1 expression, but it did lead to a decrease in SOCS2 mRNA expression (p=0.0034) and an upregulation of SOCS3 (p=0.0001). In contrast, LPS-treated decidual macrophages showed a significant upregulation in SOCS1 (p<0.0001) and SOCS3 (p=0.0005). Compared to PRL exposure, LPS treatment in both JEG-3 cells and decidual macrophages significantly enhanced SOCS expression, suggesting that SOCS1 and SOCS3 may play critical roles as negative regulators in response to LPS-induced inflammation (**Figure 4C**).

### LPS enhances the secretion of proinflammatory mediators in placental cells

**Figure 5:**
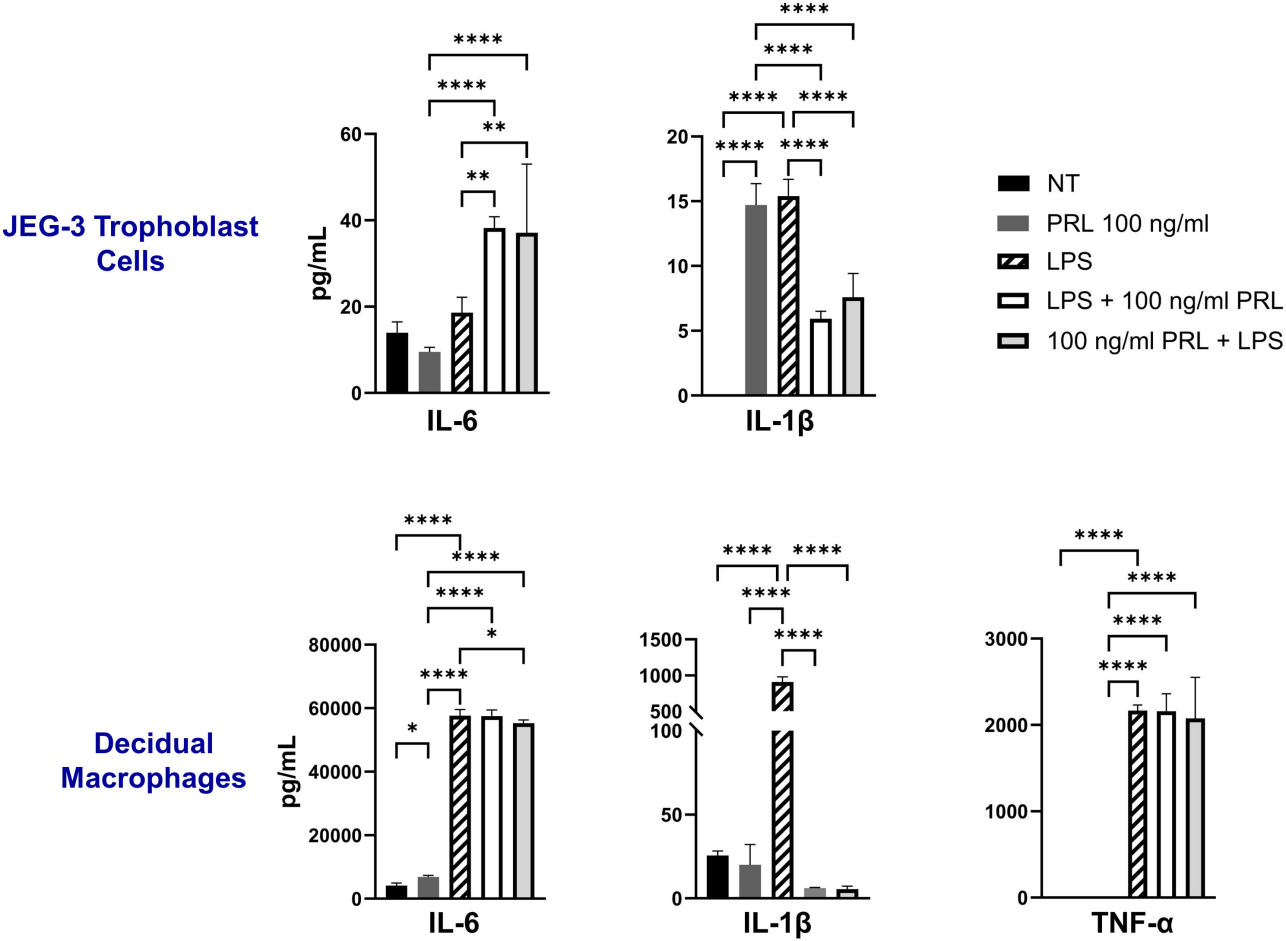
LPS upregulates PRL-receptor expression and signaling at the maternal-fetal interface. JEG-3 cells and decidual macrophages were treated with 5 μg/ml of LPS alone, treated with LPS 3 hours prior to PRL (100 ng/ml), treated with PRL (100 ng/ml) 3 hours prior to LPS, or left untreated. Quantification of inflammatory cytokines, IL-6, IL-1β, and TNF-α, were determined in the supernatants by ELISA following 48 hours of *in vitro* culture. All values are represented as pg/mL. Data shown are expressed as the mean ± standard error of biological triplicates from 8 individual donors analyzed by one-way ANOVA using Dunnett’s multiple comparisons test. ****p< 0.0001, **p <.01, and *p <.05 indicate significance between the untreated (NT) cells and the treated cells, and between the treated cells.

The LPS-induced JAK2/STAT5 gene signature we observed correlates with increased secretion of proinflammatory cytokines (IL-6, IL-1β, and TNF-α) by both decidual macrophages and JEG-3 cells. Our findings demonstrate that LPS treatment leads to elevated IL-1β secretion in both decidual macrophages and JEG-3 cells, along with increased TNF-α production in decidual macrophages. Additionally, when cells were exposed to LPS either 3 hours before or after PRL treatment, we observed a significant increase in IL-6 secretion by JEG-3 cells compared to LPS- or PRL-treated JEG-3 cells alone. In contrast, PRL significantly downregulated IL-1β secretion in JEG-3 cells and decidual macrophages co-cultured with LPS (**Figure 5**). These studies indicate that while LPS triggers prolactin activity associated with proinflammatory mediator secretion, PRL can modulate the LPS-induced immune response through distinct mechanisms.

### Differential levels of STAT5 protein activation at the maternal-fetal interface may contribute to PRL-associated inflammation

**Figure 6:**
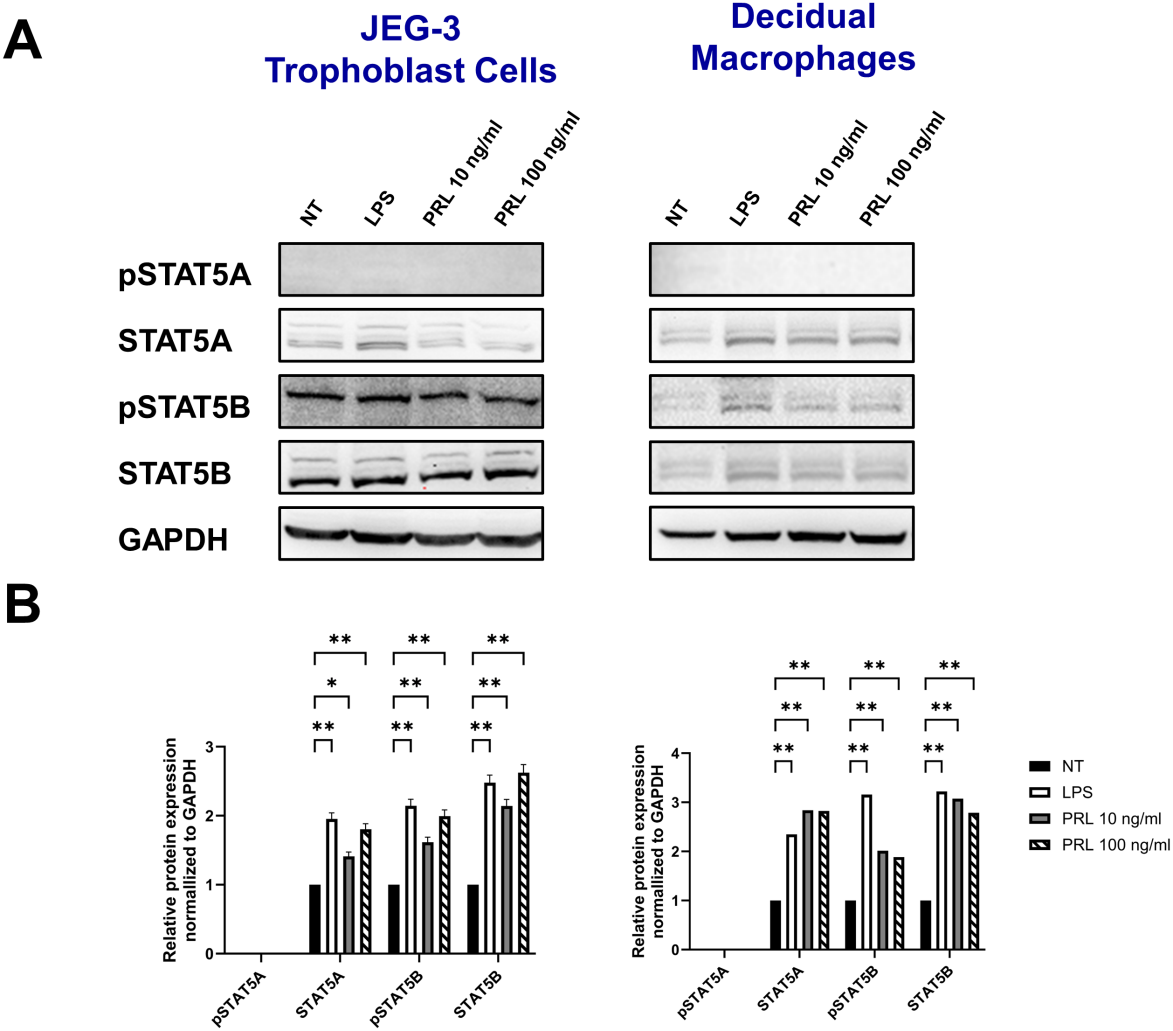
Differential levels of STAT5 protein activation at the maternal-fetal interface may contribute to PRL-associated inflammation. **(A)** JEG-3 cells and primary decidual macrophages were treated with 5 μg/ml of LPS, 10 ng/ml PRL, 100 ng/ml PRL, or left untreated for 15 minutes or 24 hours. Lysates were prepared and subjected to sodium dodecyl sulfate–polyacrylamide gel electrophoresis. The gel was blotted, and the indicated proteins were detected by immunoblotting with indicated antibodies. **(B)** The pixel intensities for pSTAT5A, STAT5A, pSTAT5B, and STAT5B of the untreated, LPS-treated and PRL-treated JEG-3 cells and decidual macrophages were quantified using ImageJ software and normalized to the GAPDH signal. Data shown are expressed as the mean ± standard error of biological triplicates from 8 individual donors analyzed by two-way ANOVA using Sidak’s multiple comparisons test. **p <.01 and *p <.05 indicate significance between the untreated (NT) cells and the LPS or PRL treated cells.

Engagement of the PRL-receptor complex triggers the activation of STAT5 by tyrosine phosphorylation. Once activated, STAT5A and STAT5B dissociate from the receptor, dimerize into either heterodimer or homodimer, and translocate to the nucleus, where they bind to the promoters of target genes, including those for pro-inflammatory cytokines. This signaling pathway remains relatively unexplored in the placenta. Our data indicate that elevated PRL levels significantly upregulate the mRNA expression of JAK2, STAT5A, and STAT5B in placental trophoblasts and decidual macrophages. Additionally, LPS appears to enhance PRL expression and PRL-induced signaling. To determine whether increased mRNA transcription translates to protein expression, we analyzed total protein levels and phosphorylation of STAT5A and STAT5B in JEG-3 cells and decidual macrophages. We found that untreated JEG-3 cells express higher levels of both total and phosphorylated STAT5B compared to decidual macrophages (**Figure 6A**). Both cell types expressed relatively low basal levels of total STAT5A and showed little to no phosphorylation of STAT5A, regardless of LPS or PRL treatment. However, following LPS treatment, total STAT5A and STAT5B protein levels were significantly upregulated in both JEG-3 cells and decidual macrophages, corresponding with a marked increase in STAT5B phosphorylation (**Figure 6B**). Similarly, PRL treatment at 10 ng/ml and 100 ng/ml significantly upregulated total STAT5A and STAT5B in both cell types, along with increased phosphorylation of STAT5B. These findings suggest that PRL signaling at the maternal-fetal interface is likely mediated via STAT5B, and its activation may play a key role in PRL-associated inflammation.

## Discussion

Pregnancy is an immunologically unique period represented by a delicate balance of endocrine and immune signals necessary to tolerate and support the development and survival of the fetus. In uncomplicated pregnancies, this balance is reflected by a Th-2 cytokine dominance. However, in pregnancies complicated by maternal infections or chronic diseases, this balance may shift, leading to an increase in the Th1:Th2 ratio, which is associated with early pregnancy loss, preeclampsia, and fetal growth restriction (3, 4). Prolactin (PRL), an essential endocrine mediator at the maternal-fetal interface, plays a critical role in immunomodulation, progesterone production, successful implantation, and fetal development (16, 39). Despite its importance, elevated maternal PRL levels have been linked to implantation failure, miscarriage, and preterm birth (40–43), however, the underlying mechanisms for these outcomes remain poorly understood. We sought to fill this knowledge gap by identifying the impact of elevated PRL on trophoblast and macrophage function at the maternal-fetal interface. Here we report that exogenous PRL triggers the JAK/STAT signaling pathway in placental trophoblasts and decidual macrophages, which induces a pro-inflammatory response, characterized by differential production of IL-1β and IL-6. Moreover, LPS exposure upregulates PRL and PRL-receptor expression, exacerbating PRL signaling and potentially triggering an inflammatory cascade at the maternal-fetal interface. Our data suggest that STAT5B activity (independent of STAT5A), plays a pivotal role in PRL- and LPS-induced inflammation. Defining mediators and mechanisms associated with placental inflammation and associated adverse pregnancy outcomes are critical to understand for the development of specific therapeutics to mitigate these consequences.

The placenta, a unique organ formed during pregnancy, regulates the intrauterine environment and serves as the master regulator of the intrauterine environment via nutrient transfer, metabolism, gas exchange, neuroendocrine signaling, growth hormone production, and immunologic surveillance (44–46). The fetal portion of the placenta is the villous chorion, and the maternal portion is the decidua basalis. Decidual stromal cells are a major source of PRL and the fetal chorionic cells express the PRL-receptor (15), suggesting a paracrine role for PRL at the maternal-fetal interface. Our findings extended these observations by demonstrating that placental trophoblast (JEG-3) upregulate PRL mRNA expression in response to exogenous PRL. This positive feedback loop has been previously demonstrated in endothelial cells (47) and autocrine production of PRL was linked to STAT5 activation (48). Additionally, the upregulation of PRL-receptor in placental trophoblasts and decidual macrophages suggests enhanced sensitivity to PRL signaling, further supporting its role as a paracrine hormone at the maternal-fetal interface. Paracrine interactions between the trophoblasts and maternal decidual have been shown to be important for successful embryonic implantation and overall maintenance of pregnancy. Recent studies suggest that that the during a healthy pregnancy, trophoblasts act to alter the local immune environment of the decidua to ensure an Th2-enriched cytokine/chemokine environment (49, 50). In addition, trophoblasts constitutively release antiviral interferons that are capable of restricting infection in both autocrine and paracrine manners (51, 52). While these intra-network communications are critical at the maternal-fetal interface, an imbalance between the local and external autocrine/paracrine mediators may lead to deleterious signaling and adverse pregnancy outcomes.

PRL is best known for its role in inducing and maintaining lactation; in addition, it regulates the function of lymphocytes, including exerting metabolic effects and stimulating immune responsiveness (53, 54). Despite the *de novo* synthesis of PRL at the maternal-fetal interface, which highlights its crucial role in pregnancy, knowledge about PRL expression and its signaling is very limited at the placenta. PRL has been shown to accumulate in maternal serum, amniotic fluid, and intervillous blood during pregnancy (55, 56) at concentrations substantially higher when compared to non-pregnant women (57). At the placenta, PRL can stimulate trophoblast migration and invasion (15) and may be key for the establishment of placental circulation, necessary for normal fetal growth and development. However, PRL may also be involved in a variety of pathological conditions related to pregnancy (40–43, 58, 59). Studies have demonstrated an increase in antiangiogenic PRL fragments in amniotic fluid and urine of women with preeclampsia (58, 59) and in diabetic placentas, a significant increase in PRL gene expression was observed (60). A positive correlation was found between increases in PRL levels in early pregnancy and subsequent risk of gestational diabetes (61). In addition, hyperprolactinemia, or high levels of prolactin in the blood, has also been linked to an increased risk of pregnancy loss, particularly those who have a history of recurrent miscarriages (10, 11). Given the association between PRL and adverse pregnancy outcomes, it is important to examine the exact molecular mechanisms involved in PRL and PRLR expression and signaling at the maternal-fetal interface.

The PRL-R is the only known receptor for PRL (17, 18) and its binding activates the JAK/STAT intracellular pathways (62); specifically JAK2, which recruits and phosphorylates STAT5. STAT5 exists in two isoforms with high sequence homology, STAT5A and STAT5B, which are encoded by two different genes. Studies have shown that STAT5A and STAT5B fulfill redundant and non-redundant functions in orchestrating immunoregulation. STAT5A alone is essential for mammary gland development and prolactin signaling (63, 64). Alternatively, STAT5B is known to mediate growth hormone signaling and mutations in the STAT5B gene have been associated with stunted growth, autoimmunity and immunodeficiency (65–69). To date, little is known of STAT5A- or STAT5B-specific functions at the placenta. Activation of STAT5A has been shown to increase over the course of pregnancy as levels of PRL rise, which may be related to lactation. Studies have also shown that the placental growth hormone (GH) can act on the JAK2/STAT5 pathway in decidual cells, which is associated with uterine receptivity for pregnancy (70). In addition, high levels of hormonal stimulation has been shown increase STAT5 expression, subsequently promoting the activation of the PRL promoter and facilitating endometrial decidualization (71). Studies in macrophages show that PRL can activate the JAK2/STAT pathway, resulting in the release of pro-inflammatory cytokines, including tumor necrosis factor alpha (TNF-α), IL-1β, IL-12, and interferon gamma (IFN-γ) (26, 72, 73). Here we demonstrate that JEG-3 cells and decidual macrophages respond robustly to PRL via the JAK2/STAT5 pathway. PRL-treated JEG-3 cells significantly upregulated JAK2, STAT5A, and STAT5B, with JAK2 and STAT5B exhibiting greater than 100-fold increases in transcription. This data supports the notion that JEG-3 cells respond robustly to exogenous PRL treatment and that JAK2 and STAT5B may be key mediators in this signaling pathway. In contrast, PRL treatment of decidual macrophages downregulated STAT5B transcription following exposure to low levels of PRL, however treatment with elevated levels of PRL induced a significant upregulation of STAT5B gene expression, along with STAT5A and JAK2. These data indicates that low levels of PRL may reduce STAT5B activity, while high levels of PRL induces transcription of STAT5A and STAT5B similarly. However, when we evaluated whether STAT5A and STAT5B transcription following PRL exposure correlated with STAT5 protein levels and activation, in the decidual macrophages and JEG-3 cells, we show that PRL treatment upregulated total protein levels of STAT5A and STAT5B similarly. Nevertheless, in both cell types, only STAT5B was phosphorylated. The impact of STAT5B activity at the maternal-fetal interface is unknown, however observations have linked aberrant activation of STAT5B to autoimmunity, the development of certain cancers, and interestingly, a syndrome of severe allergic inflammation (74, 75). Evidence also suggests that STAT5B is involved in epithelial-mesenchymal transition (EMT) of cancer cells (76). This cellular transition mechanism is a key regulator of trophoblasts invasion and placental development, however EMT dysregulation has been associated with many placental disorders (77, 78).

The SOCS proteins are well established as inhibitors of JAK/STAT function. The expression of SOCS can be induced by cytokine stimulation, and they negatively regulate JAK–STAT signals by binding to cytokine receptors and the activation loop of JAK through their central SH2 domain. SOCS1 and SOCS3 have been shown to inhibit STAT5 activation and/or target STAT5 for degradation (79–83). SOCS1 and SOCS3 can directly bind to JAK, thus inhibiting their kinase activity and ability to phosphorylate STAT5. In addition, the SOCS proteins can bind cytokine receptors, preventing the recruitment of STAT5 to the receptor complex and promotes ubiquitination of components in the JAK/STAT5 signaling pathway. Interestingly, studies show that complete absence of SOCS1 in the mammary gland leads to precocious activation of STAT5A (84). Studies have also shown that the SOCS proteins can balance STAT5 activity through the PRL-receptor. SOCS1 and SOCS3 have been shown to inhibit PRL signaling via distinct mechanisms, with SOCS3 being the most potent inhibitor (85, 86). In contrast, SOCS2 has been shown to restore PRL signal transduction that has been inhibited by SOCS1, but does not affect the SOCS3 inhibitory effect (85). Overexpression of the SOCS proteins may lead to reduced prolactin signaling, however, downregulation or mutations in SOCS genes may also result in hyperactive JAK/STAT5 signaling, promoting inflammation and unregulated cell proliferation and differentiation. For example, a recent study demonstrated that deletion of one *Socs1* alleles restored normal levels of STAT5A activity and corrected lactation failure in *Prlr* ^+/−^ mice (87). Here we show that SOCS1, SOCS2, and SOCS3 are constitutively expressed in placental cells, with decidual macrophages exhibiting overall higher gene expression levels for each protein. This basal expression may play an intrinsic protective role by limiting JAK/STAT-associated inflammation. PRL exposure significantly downregulates SOCS1 expression in decidual macrophages and JEG-3 cells and SOCS2 in JEG-3 cells. This downregulation is associated with increased JAK2/STAT5 expression and signaling. SOCS1 is a key inhibitor of PRL-STAT5 signaling, and if SOC-1 is inhibited or downregulated, unchecked PRL signaling could lead to negative consequences at the maternal-fetal interface. These results demonstrate that the SOCS proteins are differentially expressed at the maternal-fetal interface and that these proteins may serve as potential therapeutic targets in conditions where PRL signaling is dysregulated.

Inflammation at the placenta could be related to infectious or non-infectious causes, and in both cases, the resulting signaling is deleterious for pregnancy and is associated with poor fetal outcomes (88, 89). In humans, gram-negative bacterial infections are recognized as a significant cause of fetal loss and preterm birth (90, 91). Lipopolysaccharide (LPS) is a toxic component of cell walls in gram-negative bacteria and is widely used to establish bacterial infection models, including mimicking *in utero* inflammation at the maternal-fetal interface (92). In animal models, maternal LPS exposure in the first trimester results in embryonic resorption and fetal death (93, 94) and in the third trimester, maternal LPS exposure causes fetal death or preterm delivery (95). In this study, the LPS endotoxin was used to simulate an infectious scenario and to compare PRL-induced inflammation to a known inflammatory mediator. As expected, we show that LPS induces robust inflammation via JAK/STAT gene expression and downstream activation, along with secretion of inflammatory cytokines (IL-6, IL-1β, and TNF-α). Interestingly, we also show that LPS promotes enhanced PRL expression in JEG-3 cells and PRL-receptor expression in decidual macrophages, suggesting that PRL elevation may be a potential biomarker for bacterial infection at the placenta. Previous studies have demonstrated that LPS treatment of monocytes and anterior pituitary explants enhances PRL expression and secretion, and it is hypothesized that this effect of LPS could be mediated by IL-6 which is known as prolactin-releasing factor (96, 97). We also show that LPS significantly upregulates mRNA expression of SOCS1 and SOCS3 the decidual macrophages, and SOCS3 in JEG-3 cells. The SOCS have been shown to participate in negative regulation of LPS responses (98), and here we show that the SOCS may indeed be essential negative regulators of LPS signaling in the maternal decidua and chorionic villi to protect from overresponses to LPS. Overall, these data suggest that LPS exposure at the maternal-fetal interface may enhance and/or exacerbate PRL secretion and signaling, leading to aberrant inflammatory responses associated with adverse outcomes.

**Figure 7:**
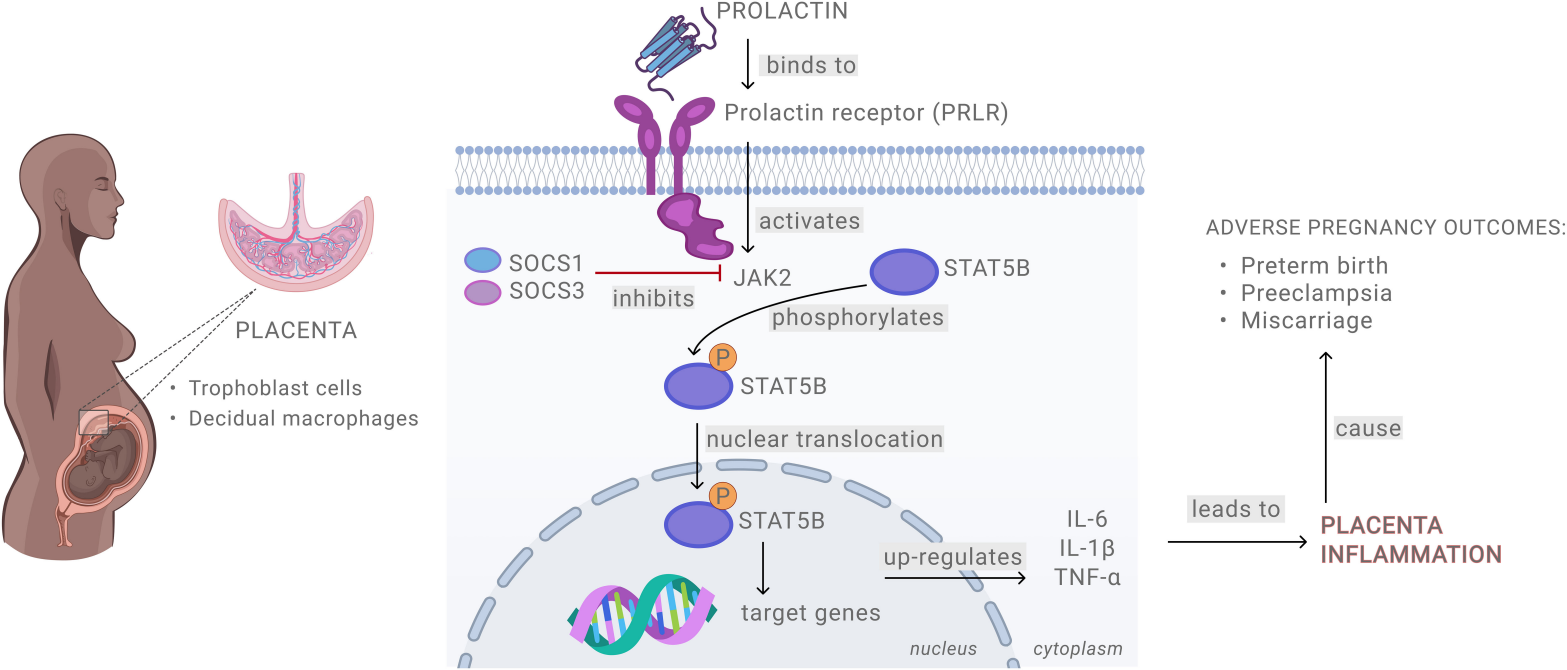
Schematic presentation of prolactin signaling at the maternal-fetal interface.

In summary, our study provides mechanistic insights into the role of PRL in placental inflammation and adverse pregnancy outcomes (**Figure 7**). We show that elevated levels of exogenous PRL activate the JAK/STAT signaling pathway in placental and decidual cells, leading to a robust pro-inflammatory response. LPS treatment further amplifies PRL signaling, potentially triggering an inflammatory cascade at the maternal-fetal interface. Notably, STAT5B appears to be a key mediator in PRL- and LPS-induced inflammation. These findings are important in defining the mediators and mechanisms driving placental inflammation and related adverse pregnancy outcomes and are critical for designing targeted therapeutics to mitigate these effects.

## Data Availability

The raw data supporting the conclusions of this article will be made available by the authors, without undue reservation.

## References

[1] Napso T Yong HEJ Lopez-Tello J Sferruzzi-Perri AN. The role of placental hormones in mediating maternal adaptations to support pregnancy and lactation. Front Physiol. (2018) 9:1091. doi: 10.3389/fphys.2018.01091 30174608 PMC6108594

[2] Wegmann TG Lin H Guilbert L Mosmann TR. Bidirectional cytokine interactions in the maternal-fetal relationship: is successful pregnancy a th2 phenomenon? Immunol Today. (1993) 14:353–6. doi: 10.1016/0167-5699(93)90235-D 8363725

[3] Negishi Y Takahashi H Kuwabara Y Takeshita T. Innate immune cells in reproduction. J Obstetrics Gynaecol Res. (2018) 44:2025–36. doi: 10.1111/jog.2018.44.issue-11 30058156

[4] Wu L Li J Xu HL Xu B Tong XH Kwak-Kim J. Il-7/il-7r signaling pathway might play a role in recurrent pregnancy losses by increasing inflammatory th17 cells and decreasing treg cells. Am J Reprod Immunol. (2016) 76:454–64. doi: 10.1111/aji.2016.76.issue-6 27767237

[5] Singh U Solanki V Mehrotra S Natu SM Chauhan S Sharma R. Study of prolactin in cervicovaginal secretion in women with preterm labor and normal pregnancy. J South Asian Fed Obstetrics Gynaecol. (2020) 12:34–7. doi: 10.5005/jp-journals-10006-1758

[6] Kaiser UB. Hyperprolactinemia and infertility: new insights. J Clin Invest. (2012) 122:3467–8. doi: 10.1172/jci64455 PMC3461927 23193578

[7] Chen H Fu J Huang W. Dopamine agonists for preventing future miscarriage in women with idiopathic hyperprolactinemia and recurrent miscarriage history. Cochrane Database Syst Rev. (2016) 7:Cd008883. doi: 10.1002/14651858.CD008883.pub2 27455388 PMC6458049

[8] Hirahara F Andoh N Sawai K Hirabuki T Uemura T Minaguchi H. Hyperprolactinemic recurrent miscarriage and results of randomized bromocriptine treatment trials. Fertility Sterility. (1998) 70:246–52. doi: 10.1016/S0015-0282(98)00164-2 9696215

[9] Ando N Gorai I Hirabuki T Onose R Hirahara F Minaguchi H. Prolactin disorders in patients with habitual abortion. Nihon Sanka Fujinka Gakkai Zasshi. (1992) 44:650–6. 1506725

[10] Hirahara F. The clinical management for recurrent spontaneous abortion. Nihon Sanka Fujinka Gakkai Zasshi. (1996) 48:169–72.

[11] Bussen S Sütterlin M Steck T. Endocrine abnormalities during the follicular phase in women with recurrent spontaneous abortion. Hum Reprod. (1999) 14:18–20. doi: 10.1093/humrep/14.1.18 10374087

[12] Ben-Jonathan N Mershon JL Allen DL Steinmetz RW. Extrapituitary prolactin: distribution, regulation, functions, and clinical aspects. Endocr Rev. (1996) 17:639–69. doi: 10.1210/edrv-17-6-639 8969972

[13] Aguilar-Rojas A Huerta-Reyes M. Human gonadotropin-releasing hormone receptor-activated cellular functions and signaling pathways in extra-pituitary tissues and cancer cells. Oncol Rep. (2009) 22:981–90. doi: 10.3892/or_00000525 19787210

[14] Gorvin CM. The prolactin receptor: diverse and emerging roles in pathophysiology. J Clin Transl Endocrinol. (2015) 2:85–91. doi: 10.1016/j.jcte.2015.05.001 29204371 PMC5685068

[15] Stefanoska I Jovanović Krivokuća M Vasilijić S Ćujić D Vićovac L. Prolactin stimulates cell migration and invasion by human trophoblast. In Vitro Placenta. (2013) 34:775–83. doi: 10.1016/j.placenta.2013.06.305 23849393

[16] Binart M. Prolactin and pregnancy in mice and humans. Ann Endocrinol (Paris). (2017) 77(2):126-7. doi: 10.1016/j.ando.2016.04.008 27155773

[17] Bole-Feysot C Goffin V Edery M Binart N Kelly PA. Prolactin (Prl) and its receptor: actions, signal transduction pathways and phenotypes observed in prl receptor knockout mice. Endocrine Rev. (1998) 19:225–68. doi: 10.1210/edrv.19.3.0334 9626554

[18] Clevenger CV Gadd SL Zheng J. New mechanisms for prlr action in breast cancer. Trends Endocrinol Metab. (2009) 20:223–9. doi: 10.1016/j.tem.2009.03.001 19535262

[19] Gong N Ferreira-Martins D McCormick SD Sheridan MA. Divergent genes encoding the putative receptors for growth hormone and prolactin in sea lamprey display distinct patterns of expression. Sci Rep. (2020) 10:1674. doi: 10.1038/s41598-020-58344-5 32015405 PMC6997183

[20] Aoki M Wartenberg P Grünewald R Phillipps HR Wyatt A Grattan DR. Widespread cell-specific prolactin receptor expression in multiple murine organs. Endocrinology. (2019) 160:2587–99. doi: 10.1210/en.2019-00234 31373638

[21] Pezet A Buteau H Kelly PA Edery M. The last proline of box 1 is essential for association with jak2 and functional activation of the prolactin receptor. Mol Cell Endocrinol. (1997) 129:199–208. doi: 10.1016/S0303-7207(97)00063-4 9202403

[22] DaSilva L Rui H Erwin RA Howard OZ Kirken RA Malabarba MG. Prolactin recruits stat1, stat3 and stat5 independent of conserved receptor tyrosines tyr402, tyr479, tyr515 and tyr580. Mol Cell Endocrinol. (1996) 117:131–40. doi: 10.1016/0303-7207(95)03738-1 8737372

[23] Abramicheva P Smirnova O. Prolactin receptor isoforms as the basis of tissue-specific action of prolactin in the norm and pathology. Biochem (Moscow). (2019) 84:329–45. doi: 10.1134/S0006297919040011 31228925

[24] Bouilly J Sonigo C Auffret J Gibori G Binart N. Prolactin signaling mechanisms in ovary. Mol Cell Endocrinol. (2012) 356:80–7. doi: 10.1016/j.mce.2011.05.004 21664429

[25] Shuai K. Regulation of cytokine signaling pathways by pias proteins. Cell Res. (2006) 16:196–202. doi: 10.1038/sj.cr.7310027 16474434

[26] Sodhi A Tripathi A. Prolactin and growth hormone induce differential cytokine and chemokine profile in murine peritoneal macrophages *in vitro*: involvement of P-38 map kinase, stat3 and nf-κb. Cytokine. (2008) 41:162–73. doi: 10.1016/j.cyto.2007.11.007 18248819

[27] Zaga-Clavellina V Parra-Covarrubias A Ramirez-Peredo J Vega-Sanchez R Vadillo-Ortega F. The potential role of prolactin as a modulator of the secretion of proinflammatory mediators in chorioamniotic membranes in term human gestation. Am J Obstetrics Gynecol. (2014) 211:48.e1–e6. doi: 10.1016/j.ajog.2014.01.039 24495670

[28] Flores-Espinosa P Preciado-Martínez E Mejía-Salvador A Sedano-González G Bermejo-Martínez L Parra-Covarruvias A. Selective immuno-modulatory effect of prolactin upon pro-inflammatory response in human fetal membranes. J Reprod Immunol. (2017) 123:58–64. doi: 10.1016/j.jri.2017.09.004 28938125

[29] Tomio A Schust DJ Kawana K Yasugi T Kawana Y Mahalingaiah S. Prolactin can modulate cd4+ T-cell response through receptor-mediated alterations in the expression of T-bet. Immunol Cell Biol. (2008) 86:616–21. doi: 10.1038/icb.2008.29 18414429

[30] Mackern-Oberti JP Jara EL Riedel CA Kalergis AM. Hormonal modulation of dendritic cells differentiation, maturation and function: implications for the initiation and progress of systemic autoimmunity. Archivum Immunol Ther Experimentalis. (2017) 65:123–36. doi: 10.1007/s00005-016-0418-6 27585815

[31] Carreño PC Jiménez E Sacedón R Vicente A. Zapata anG. Prolactin stimulates maturation and function of rat thymic dendritic cells. J Neuroimmunol. (2004) 153:83–90. doi: 10.1016/j.jneuroim.2004.04.020 15265666

[32] Borba VV Zandman-Goddard G Shoenfeld Y. Prolactin and autoimmunity. Front Immunol. (2018) 9:73. doi: 10.3389/fimmu.2018.00073 29483903 PMC5816039

[33] Johnson EL Chakraborty R. Placental hofbauer cells limit hiv-1 replication and potentially offset mother to child transmission (Mtct) by induction of immunoregulatory cytokines. Retrovirology. (2012) 9:101. doi: 10.1186/1742-4690-9-101 23217137 PMC3524025

[34] Schindelin J Arganda-Carreras I Frise E Kaynig V Longair M Pietzsch T. Fiji: an open-source platform for biological-image analysis. Nat Methods. (2012) 9:676–82. doi: 10.1038/nmeth.2019 PMC3855844 22743772

[35] Benjamini Y Hochberg Y. Controlling the false discovery rate: A practical and powerful approach to multiple testing. J R Stat Soc Ser B (Methodological). (1995) 57:289–300. doi: 10.1111/j.2517-6161.1995.tb02031.x

[36] Luo W Li YX Jiang LJ Chen Q Wang T Ye DW. Targeting jak-stat signaling to control cytokine release syndrome in covid-19. Trends Pharmacol Sci. (2020) 41:531–43. doi: 10.1016/j.tips.2020.06.007 PMC7298494 32580895

[37] De Laurentiis A Pisera D Caruso C Candolfi M Mohn C Rettori V. Lipopolysaccharide- and tumor necrosis factor-alpha-induced changes in prolactin secretion and dopaminergic activity in the hypothalamic-pituitary axis. Neuroimmunomodulation. (2002) 10:30–9. doi: 10.1159/000064412 12207161

[38] Gilstrap LC 3rd Ramin SM. Urinary tract infections during pregnancy. Obstet Gynecol Clin North Am. (2001) 28:581–91. doi: 10.1016/s0889-8545(05)70219-9 11512502

[39] Soares MJ Konno T Alam SK. The prolactin family: effectors of pregnancy-dependent adaptations. Trends Endocrinol Metab. (2007) 18:114–21. doi: 10.1016/j.tem.2007.02.005 17324580

[40] Garzia E Clauser R Persani L Borgato S Bulfamante G Avagliano L. Prolactin and proinflammatory cytokine expression at the fetomaternal interface in first trimester miscarriage. Fertility Sterility. (2013) 100:108–15. doi: 10.1016/j.fertnstert.2013.02.053 23541403

[41] Garzia E Borgato S Cozzi V Doi P Bulfamante G Persani L. Lack of expression of endometrial prolactin in early implantation failure: A pilot study. Hum Reprod. (2004) 19:1911–6. doi: 10.1093/humrep/deh350 15218000

[42] Luciano AA Varner MW. Decidual, amniotic fluid, maternal and fetal prolactin in normal and abnormal pregnancies. Obstetrics Gynecol. (1984) 63:384–8. 6366659

[43] Grosdemouge I Bachelot A Lucas A Baran N Kelly PA Binart N. Effects of deletion of the prolactin receptor on ovarian gene expression. Reprod Biol Endocrinol. (2003) 1:1–16. doi: 10.1186/1477-7827-1-12 PMC151786 12646063

[44] McKay R. Developmental biology: remarkable role for the placenta. Nature. (2011) 472:298–9. doi: 10.1038/472298a 21512563

[45] Thornburg KL O’Tierney PF Louey S. Review: the placenta is a programming agent for cardiovascular disease. Placenta. (2010) 31 Suppl:S54–9. doi: 10.1016/j.placenta.2010.01.002 PMC2846089 20149453

[46] Benirschke K Driscoll SG. The Pathology of the Human Placenta. New York, NY: Springer (1967).

[47] Yang X Friedl A. A positive feedback loop between prolactin and stat5 promotes angiogenesis. Adv Exp Med Biol. (2015) 846:265–80. doi: 10.1007/978-3-319-12114-7_12 25472543

[48] Yang X Meyer K Friedl A. Stat5 and prolactin participate in a positive autocrine feedback loop that promotes angiogenesis. J Biol Chem. (2013) 288:21184–96. doi: 10.1074/jbc.M113.481119 PMC3774384 23729680

[49] Hess AP Hamilton AE Talbi S Dosiou C Nyegaard M Nayak N. Decidual stromal cell response to paracrine signals from the trophoblast: amplification of immune and angiogenic modulators1. Biol Reprod. (2007) 76:102–17. doi: 10.1095/biolreprod.106.054791 17021345

[50] Hess AP Hamilton AE Dosiu C Talbi S Gembacer-Krtolica O Giudice LC. A functional genomics approach to elucidate paracrine interactions between the human trophoblast and maternal decidua. Fertility Sterility. (2005) 84:S8. doi: 10.1016/j.fertnstert.2005.07.008

[51] Bayer A Lennemann NJ Ouyang Y Bramley JC Morosky S Marques ET Jr.. Type iii interferons produced by human placental trophoblasts confer protection against zika virus infection. Cell Host Microbe. (2016) 19:705–12. doi: 10.1016/j.chom.2016.03.008 PMC4866896 27066743

[52] Corry J Arora N Good CA Sadovsky Y Coyne CB. Organotypic models of type iii interferon-mediated protection from zika virus infections at the maternal–fetal interface. Proc Natl Acad Sci. (2017) 114:9433–8. doi: 10.1073/pnas.1707513114 PMC5584447 28784796

[53] Tyson J Friesen H. Factors influencing the secretion of human prolactin and growth hormone in menstrual and gestational women. Am J Obstetrics Gynecol. (1973) 116:377–87. doi: 10.1016/S0002-9378(15)31297-7 4196484

[54] Majumdar A Mangal NS. "Hyperprolactinemia". In: Ghumman S, editor. Principles and Practice of Controlled Ovarian Stimulation in Art. New Delhi: Springer India (2015). p. 319-28.

[55] Soares MJ Faria TN Roby KF Deb S. Pregnancy and the prolactin family of hormones: coordination of anterior pituitary, uterine, and placental expression. Endocr Rev. (1991) 12:402–23. doi: 10.1210/edrv-12-4-402 1760995

[56] Zhang F Xia H Shen M Li X Qin L Gu H. Are prolactin levels linked to suction pressure? Breastfeeding Med. (2016) 11:461–8. doi: 10.1089/bfm.2015.0083 27643921

[57] Laboratories MC. Mayo-clinic-laboratories prolactin serum reference values (2018). Available online at: https://endocrinology.testcatalog.org/show/PRL. (accessed July 19, 2024).

[58] Leanos-Miranda A Márquez-Acosta J Cardenas-Mondragon GM Chinolla-Arellano ZL Rivera-Leanos R Bermejo-Huerta S. Urinary prolactin as a reliable marker for preeclampsia, its severity, and the occurrence of adverse pregnancy outcomes. J Clin Endocrinol Metab. (2008) 93:2492–9. doi: 10.1210/jc.2008-0305 18460570

[59] Masumoto A Masuyama H Takamoto N Akahori Y Hiramatsu Y. Expression of antiangiogenic prolactin fragments in the placentas of women with pregnancy induced hypertension. Acta Med Okayama. (2010) 64:249–55. doi: 10.18926/AMO/40133 20802542

[60] Perimenis P Bouckenooghe T Delplanque J Moitrot E Eury E Lobbens S. Placental antiangiogenic prolactin fragments are increased in human and rat maternal diabetes. Biochim Biophys Acta (BBA)-Molecular Basis Dis. (2014) 1842:1783–93. doi: 10.1016/j.bbadis.2014.06.026 24984282

[61] Li M Song Y Rawal S Hinkle SN Zhu Y Tekola-Ayele F. Plasma prolactin and progesterone levels and the risk of gestational diabetes: A prospective and longitudinal study in a multiracial cohort. Front Endocrinol. (2020) 11:83. doi: 10.3389/fendo.2020.00083 PMC7058109 32180760

[62] Brooks CL. Molecular mechanisms of prolactin and its receptor. Endocrine Rev. (2012) 33:504–25. doi: 10.1210/er.2011-1040 PMC3410225 22577091

[63] Liu X Robinson GW Wagner K-U Garrett L Wynshaw-Boris A Hennighausen L. Stat5a is mandatory for adult mammary gland development and lactogenesis. Genes Dev. (1997) 11:179–86. doi: 10.1101/gad.11.2.179 9009201

[64] Yamaji D Na R Feuermann Y Pechhold S Chen W Robinson GW. Development of mammary luminal progenitor cells is controlled by the transcription factor stat5a. Genes Dev. (2009) 23:2382–7. doi: 10.1101/gad.1840109 PMC2764497 19833766

[65] LIu X Robinson GW Gouilleux F Groner B Hennighausen L. Cloning and expression of stat5 and an additional homologue (Stat5b) involved in prolactin signal transduction in mouse mammary tissue. Proc Natl Acad Sci. (1995) 92:8831–5. doi: 10.1073/pnas.92.19.8831 PMC41061 7568026

[66] Udy GB Towers RP Snell RG Wilkins RJ Park SH Ram PA. Requirement of stat5b for sexual dimorphism of body growth rates and liver gene expression. Proc Natl Acad Sci U.S.A. (1997) 94:7239–44. doi: 10.1073/pnas.94.14.7239 PMC23803 9207075

[67] Hwa V. Stat5b deficiency: impacts on human growth and immunity. Growth Hormone IGF Res. (2016) 28:16–20. doi: 10.1016/j.ghir.2015.12.006 PMC4846566 26703237

[68] Hwa V. Human growth disorders associated with impaired gh action: defects in stat5b and jak2. Mol Cell Endocrinol. (2021) 519:111063. doi: 10.1016/j.mce.2020.111063 33122102 PMC7736371

[69] Hwa V Nadeau K Wit JM Rosenfeld RG. Stat5b deficiency: lessons from stat5b gene mutations. Best Pract Res Clin Endocrinol Metab. (2011) 25:61–75. doi: 10.1016/j.beem.2010.09.003 21396575

[70] Cui N Li A-M Luo Z-Y Zhao Z-M Xu Y-M Zhang J. Effects of growth hormone on pregnancy rates of patients with thin endometrium. J Endocrinol Invest. (2019) 42:27–35. doi: 10.1007/s40618-018-0877-1 29671256

[71] Mak I Brosens J Christian M Hills F Chamley L Regan L. Regulated expression of signal transducer and activator of transcription, stat5, and its enhancement of prl expression in human endometrial stromal cells *in vitro* . J Clin Endocrinol Metab. (2002) 87:2581–8. doi: 10.1210/jcem.87.6.8576 12050218

[72] Tripathi A Sodhi A. Prolactin-induced production of cytokines in macrophages *in vitro* involves jak/stat and jnk mapk pathways. Int Immunol. (2008) 20:327–36. doi: 10.1093/intimm/dxm145 18187558

[73] Legorreta-Haquet MV Santana-Sánchez P Chávez-Sánchez L Chávez-Rueda AK. The effect of prolactin on immune cell subsets involved in sle pathogenesis. Front Immunol. (2022) 13:1016427. doi: 10.3389/fimmu.2022.1016427 36389803 PMC9650038

[74] Ma CA Xi L Cauff B DeZure A Freeman AF Hambleton S. Somatic stat5b gain-of-function mutations in early onset nonclonal eosinophilia, urticaria, dermatitis, and diarrhea. Blood J Am Soc Hematol. (2017) 129:650–3. doi: 10.1182/blood-2016-09-737817 PMC5290989 27956386

[75] Rani A Murphy JJ. Stat5 in cancer and immunity. J Interferon Cytokine Res. (2016) 36:226–37. doi: 10.1089/jir.2015.0054 26716518

[76] Lee TK Man K Poon RT Lo CM Yuen AP Ng IO. Signal transducers and activators of transcription 5b activation enhances hepatocellular carcinoma aggressiveness through induction of epithelial-mesenchymal transition. Cancer Res. (2006) 66:9948–56. doi: 10.1158/0008-5472.CAN-06-1092 17047057

[77] Zhu JY Pang ZJ Yu YH. Regulation of trophoblast invasion: the role of matrix metalloproteinases. Rev Obstet Gynecol. (2012) 5:e137–43. PMC3594863 23483768

[78] Redman CW Sargent IL. Latest advances in understanding preeclampsia. Science. (2005) 308:1592–4. doi: 10.1126/science.1111726 15947178

[79] Piessevaux J Lavens D Montoye T Wauman J Catteeuw D Vandekerckhove J. Functional cross-modulation between socs proteins can stimulate cytokine signaling. J Biol Chem. (2006) 281:32953–66. doi: 10.1074/jbc.M600776200 16956890

[80] Yoshimura A Naka T Kubo M. Socs proteins, cytokine signalling and immune regulation. Nat Rev Immunol. (2007) 7:454–65. doi: 10.1038/nri2093 17525754

[81] Linossi EM Nicholson SE. The socs box-adapting proteins for ubiquitination and proteasomal degradation. IUBMB Life. (2012) 64:316–23. doi: 10.1002/iub.1011 22362562

[82] Croker BA Kiu H Nicholson SE. Socs regulation of the jak/stat signalling pathway. Semin Cell Dev Biol. (2008) 19:414–22. doi: 10.1016/j.semcdb.2008.07.010 PMC2597703 18708154

[83] Tamiya T Kashiwagi I Takahashi R Yasukawa H Yoshimura A. Suppressors of cytokine signaling (Socs) proteins and jak/stat pathways: regulation of T-cell inflammation by socs1 and socs3. Arterioscler Thromb Vasc Biol. (2011) 31:980–5. doi: 10.1161/atvbaha.110.207464 21508344

[84] Harris J Stanford PM Sutherland K Oakes SR Naylor MJ Robertson FG. Socs2 and elf5 mediate prolactin-induced mammary gland development. Mol Endocrinol. (2006) 20:1177–87. doi: 10.1210/me.2005-0473 16469767

[85] Pezet A Favre H Kelly PA Edery M. Inhibition and restoration of prolactin signal transduction by suppressors of cytokine signaling*. J Biol Chem. (1999) 274:24497–502. doi: 10.1074/jbc.274.35.24497 10455112

[86] Nicola NA Greenhalgh CJ. The suppressors of cytokine signaling (Socs) proteins: important feedback inhibitors of cytokine action. Exp Hematol. (2000) 28:1105–12. doi: 10.1016/S0301-472X(00)00525-7 11027828

[87] Lindeman GJ Wittlin S Lada H Naylor MJ Santamaria M Zhang J-G. Socs1 deficiency results in accelerated mammary gland development and rescues lactation in prolactin receptor–deficient mice. Genes Dev. (2001) 15:1631–6. doi: 10.1101/gad.880801 PMC312725 11445538

[88] Tchirikov M Schlabritz-Loutsevitch N Maher J Buchmann J Naberezhnev Y Winarno AS. Mid-trimester preterm premature rupture of membranes (Pprom): etiology, diagnosis, classification, international recommendations of treatment options and outcome. J Perinat Med. (2018) 46:465–88. doi: 10.1515/jpm-2017-0027 28710882

[89] Romero R Miranda J Chaiworapongsa T Korzeniewski SJ Chaemsaithong P Gotsch F. Prevalence and clinical significance of sterile intra-amniotic inflammation in patients with preterm labor and intact membranes. Am J Reprod Immunol. (2014) 72:458–74. doi: 10.1111/aji.12296 PMC4192099 25078709

[90] Romero R Roslansky P Oyarzun E Wan M Emamian M Novitsky TJ. Labor and infection: ii. Bacterial endotoxin in amniotic fluid and its relationship to the onset of preterm labor. Am J Obstetrics Gynecol. (1988) 158:1044–9. doi: 10.1016/0002-9378(88)90216-5 3369483

[91] Hazan Y Mazor M Horowitz S Leiberman JR Glezerman M. The diagnostic value of amniotic fluid gram stain examination and limulus amebocyte lysate assay in patients with preterm birth. Acta Obstetricia Gynecol Scandinavica. (1995) 74:275–80. doi: 10.3109/00016349509024449 7537430

[92] Olmos-Ortiz A Déciga-García M Preciado-Martínez E Bermejo-Martínez L Flores-Espinosa P Mancilla-Herrera I. Prolactin decreases lps-induced inflammatory cytokines by inhibiting tlr-4/nfκb signaling in the human placenta. Mol Hum Reprod. (2019) 25:660–7. doi: 10.1093/molehr/gaz038 PMC6821386 31263869

[93] Aisemberg J Vercelli C Billi S Ribeiro M Ogando D Meiss R. Nitric oxide mediates prostaglandins’ Deleterious effect on lipopolysaccharide-triggered murine fetal resorption. Proc Natl Acad Sci. (2007) 104:7534–9. doi: 10.1073/pnas.0702279104 PMC1863444 17460035

[94] Ogando D Paz D Cella M Franchi A. The fundamental role of increased production of nitric oxide in lipopolysaccharide-induced embryonic resorption in mice. Reproduction. (2003) 125:95–110. doi: 10.1530/rep.0.1250095 12622700

[95] Leazer TM Barbee B Ebron-McCoy M Henry-Sam GA Rogers JM. Role of the maternal acute phase response and tumor necrosis factor alpha in the developmental toxicity of lipopolysaccharide in the cd-1 mouse. Reprod Toxicol. (2002) 16:173–9. doi: 10.1016/S0890-6238(02)00011-4 11955948

[96] López-Rincón G Pereira-Suárez AL Del Toro-Arreola S Sánchez-Hernández PE Ochoa-Zarzosa A Muñoz-Valle JF. Lipopolysaccharide induces the expression of an autocrine prolactin loop enhancing inflammatory response in monocytes. J Inflammation. (2013) 10:1–12. doi: 10.1186/1476-9255-10-24 PMC3716533 23731754

[97] Tomaszewska-Zaremba D Haziak K Tomczyk M Herman AP. Inflammation and lps-binding protein enable the stimulatory effect of endotoxin on prolactin secretion in the ovine anterior pituitary: ex vivo study. Mediators Inflammation. (2018) 2018:5427089. doi: 10.1155/2018/5427089 PMC6112077 30186037

[98] Nakagawa R Naka T Tsutsui H Fujimoto M Kimura A Abe T. Socs-1 participates in negative regulation of lps responses. Immunity. (2002) 17:677–87. doi: 10.1016/S1074-7613(02)00449-1 12433373

