# Supplementary material for: Elevated levels of exogenous prolactin promote inflammation at the maternal-fetal interface via the JAK2/STAT5B signaling axis
Source: Front Immunol. 2024 Dec 23;15:1496610. doi: 10.3389/fimmu.2024.1496610 (PMC11701216; doi:10.3389/fimmu.2024.1496610)
Supplement: Supplementary file 1 [file DataSheet1.pdf]

S1 Table. Primer Sequences for qRT-PCR

Gene	Forward Primer (5'- 3')	Reverse Primer (5'- 3')
β-ACTIN	GGCCCAGTCCTCCCCAAGTCCAC	GGTAAGCCCTGGCTGCCTCCACC
PRL	GAGGAGCAAACCAAACGGCTTC	AAGGCGAGACTCTTCATCAGCC
PRL receptor	CATGGTGACCTGCATCTTTCCG	GTGGGAGGAAAGTCTTGGCATC
JAK2	CCAGATGGAAACTGTTCGCTCAG	GAGGTTGGTACATCAGAAACACC
STAT5A	GTTCAGTGTTGGCAGCAATGAGC	AGCACAGTAGCCGTGGCATTGT
STAT5B	GCCACTGTTCTCTGGGACAATG	ACACGAGGTTCTCCTTGGTCAG
SOCS1	TTCGCCCTTAGCGTGAAGATGG	TAGTGCTCCAGCAGCTCGAAGA
SOCS2	GGTCGGCGGAGGAGCCATCC	GAAAGTTCCTTCTGGTGCCTCTT
SOCS3	CATCTCTGTCGGAAGACCGTCA	GCATCGTACTGGTCCAGGAACT
