# Supplementary material for: Elevated levels of exogenous prolactin promote inflammation at the maternal-fetal interface via the JAK2/STAT5B signaling axis
Source: Front Immunol. 2024 Dec 23;15:1496610. doi: 10.3389/fimmu.2024.1496610 (PMC11701216; doi:10.3389/fimmu.2024.1496610)
Supplement: Supplementary file 2 [file DataSheet2.pdf]

S2 Table. Description of antibodies used in western blot experiments.

Antibody	Host	Provider	Catalog number / RRID	Dilution
STAT5A	Rabbit	Proteintech, Rosemont, IL	13179-1-AP	1:1000
pSTAT5A	Rabbit	Proteintech	80115-1-RR	1:5000
STAT5B	Rabbit	Proteintech	51072-2-AP	1:1500
pSTAT5B	Rabbit	Abcam, Cambridge, UK	ab52211	1:1000
GAPDH	Rabbit	Cell Signaling, Danvers, MA	5174S	1:1000
