# Supplementary figures and images for: Elevated levels of exogenous prolactin promote inflammation at the maternal-fetal interface via the JAK2/STAT5B signaling axis
Source: Front Immunol. 2024 Dec 23;15:1496610. doi: 10.3389/fimmu.2024.1496610 (PMC11701216; doi:10.3389/fimmu.2024.1496610)

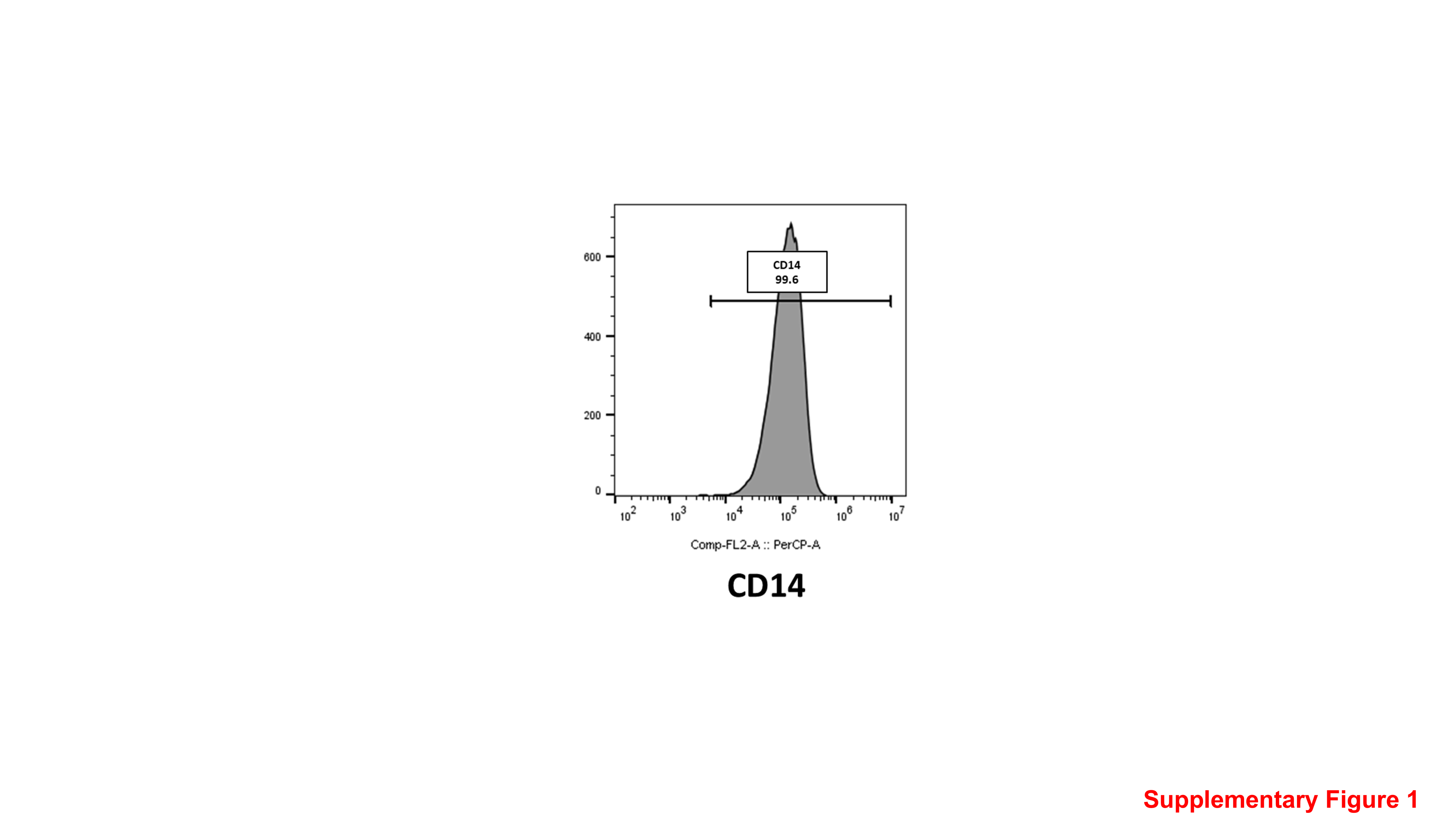

Supplement: Supplementary file 3 [file Image1.tif]
